# Combine Surgery and In Vitro Fertilization (IVF) in Endometriosis-Related Infertility: When and Why

**DOI:** 10.3390/jcm13237349

**Published:** 2024-12-02

**Authors:** Irene Colombi, Alessandro Ginetti, Alberto Cannoni, Giulia Cimino, Claudia d’Abate, Giorgia Schettini, Matteo Giorgi, Diego Raimondo, Francesco Giuseppe Martire, Lucia Lazzeri, Errico Zupi, Gabriele Centini

**Affiliations:** 1 Department of Molecular and Developmental Medicine, Obstetrics and Gynecological Clinic University of Siena, 51300 Siena, Italy; colombi.irene1@gmail.com (I.C.); ginettialessandro14@gmail.com (A.G.); albertoacannoni@gmail.com (A.C.); giulia.cimino@student.unisi.it (G.C.); claudiadabate94@gmail.com (C.d.); giorgiaschettini@gmail.com (G.S.); francesco.martire@ao-siena.toscana.it (F.G.M.); errico.zupi@unisi.it (E.Z.); centini.gabriele@gmail.com (G.C.); 2 Obstetrics and Gynecology Unit, Valdarno Hospital, 52025 Montevarchi, Arezzo, Italy; matteogiorgi@hotmail.it; 3 Division of Gynaecology and Human Reproduction Physiopathology, Department of Medical and Surgical Sciences (DIMEC), IRCCS Azienda Ospedaliera Univeristaria di Bologna. S. Orsola Hospital, University of Bologna, 40126 Bologna, Italy; die.raimondo@gmail.com

**Keywords:** endometriosis, infertility, in vitro fertilization

## Abstract

Endometriosis is a chronic, estrogen-dependent inflammatory disease characterized by the presence of endometrial tissue outside the uterus, causing pelvic pain and infertility. Infertility arises mainly due to inflammatory mediators in the peritoneal fluid, contributing to local hypoestrogenism, which appears to exacerbate chronic inflammation and sensitize pelvic nerves. Local hypoestrogenism within endometriotic lesions contrasts with the systemic estrogen-dependent nature of the disease. This localized reduction in estrogen levels, resulting from an altered hormonal response, can contribute to the altered immune response and inflammation characteristic of endometriosis, potentially exacerbating tissue damage, promoting fibrosis, adhesions, and endometrioma formation that distort pelvic anatomy, and affecting fertility. Chronic pelvic pain and dyspareunia further complicate conception in affected women. In vitro fertilization (IVF) and laparoscopic surgical excision of endometriotic lesions are the two primary management options for endometriosis-related infertility, although current data provide limited guidance on when to prefer one approach over the other. It is generally accepted that treatment strategies must be individualized according to the patient’s wishes, symptomatology, age and the preferences of the woman and the couple. Timely intervention and structured follow-up for symptomatic women wishing to conceive may maximize conception rates within two years post-surgery, while minimizing the need for repeated interventions, which should be avoided. On the other hand, first-line IVF is particularly viable in cases of unoperated deep infiltrating endometriosis in asymptomatic women, or for those ineligible for or opposed to surgery. This review aims to evaluate the most recent data on endometriosis-related infertility to identify evidence-based key points that can enhance tailored management in clinical practice.

## 1. Introduction

Endometriosis is a multifactorial disease characterized by the presence of endometrial glands and stroma outside the uterine cavity [1], which is often associated with chronic pelvic pain and adverse reproductive outcomes through several direct and indirect mechanisms [2]. Various theories have been proposed to explain the pathogenesis of endometriosis, including retrograde menstruation, coelomic metaplasia, lymphatic or vascular spread, immune system dysfunction, genetic factors, and environmental influences. These theories are not mutually exclusive, a combination of factors contributes to the development of endometriosis [3]. However, all proposed theories share a common underlying factor: a complex dysregulation of hormonal signaling characterized by local hypoestrogenism and progesterone resistance and an enhanced proinflammatory microenvironment. These two key biological mechanisms contribute to a sequence of bleeding, inflammation, fibrin deposition, adhesion formation, scarring and distortion of peritoneal surfaces of organs and pelvic anatomy, which drive the natural history of this disease [2,3,4].

Infertility, defined as the inability to conceive after twelve or more months prior to examination [5], is one of the main symptoms of endometriosis and sometimes the only one. The main mechanisms leading to infertility are the presence of inflammatory mediators in the peritoneal fluid, which can affect the release of the oocyte and its quality, the development of adhesions, which disrupt the pelvic anatomy and cause loss of tubal function, the presence of endometriomas (OMAs) affecting folliculogenesis [6] and an impaired endometrial receptivity [2]. OMAs show a detrimental effect on ovarian function. However, the exact pathophysiological mechanism responsible for the diminished ovarian reserve observed in endometriosis remains elusive. This phenomenon may arise from mechanical stretching damage or direct toxic effects. Notably, endometriotic cyst fluid is rich in free iron, which triggers the production of reactive oxygen species (ROS). Consequently, oxidative stress occurs in the viable cells of the normal ovarian cortex, leading to fibrosis, inhibition of angiogenesis, and follicular damage [7]. Early follicles may undergo atresia due to these processes [8]. Ovarian reserve, clinically evaluated by antral follicle count (AFC) and serum anti-Mullerian hormone levels (AMHs) [9], is a pivotal factor to evaluate a patient’s reproductive chances, closely related to a woman’s age [2]. Therefore, the average delay of 7 years to diagnosis of endometriosis is particularly critical for women facing infertility, as the combined effects of endometriosis and advancing age on ovarian reserve can severely compromise a woman’s chances of becoming pregnant [10,11].

In addition, chronic pelvic pain and both superficial and deep dyspareunia associated with endometriosis may contribute to difficulties in conceiving. Superficial dyspareunia, characterized by pain in or around the vaginal introitus, can hinder intercourse initiation, while deep dyspareunia, often linked to the infiltrative form of endometriosis affecting pelvic structures, such as the posterior vaginal fornix, the pouch of Douglas, the uterosacral ligaments, and the rectum [12,13,14], can make intercourse difficult to sustain. As a result, sexual dysfunction may occur, impacting desire, frequency of intercourse, arousal, and orgasm, thus significantly affecting intimate relationships and overall quality of life. Therefore, a holistic approach, rather than a purely mechanistic one, is essential [15,16]. Choosing the optimal treatment for infertility in patients with endometriosis is still a challenge: surgeons and medically assisted reproductive physicians try to further their cause, so the debate about surgical treatment or in vitro fertilization (IVF) is still open. The aim of this review is to analyze the different points of view and to provide decision-making tools to offer the most appropriate treatment, particularly the role of surgery in treating infertility, although it is not the only therapeutic strategy, as other options such as lipiodol flushing are also reported in the literature, in selected patients [17]. Furthermore, by excluding adenomyosis, which, although closely associated, is a separate clinic entity, we likely aimed to maintain a clear focus on endometriosis specifically affecting the pelvis and its reproductive implications, allowing for a more streamlined discussion of management strategies related to fertility and the various presentations of endometriosis.

## 2. Search Methodology

**Figure 1:**
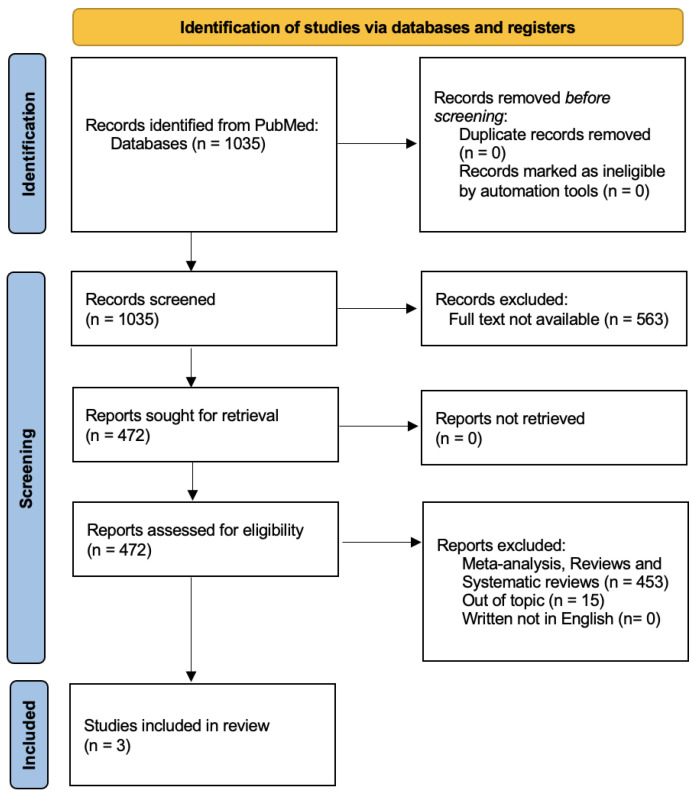
PRISMA 2020 flow diagram which includes searches of PubMed. Literature search diagram. A total of 1035 papers filled the search string. Of these, 563 articles were excluded because the full text was not available. In addition, 453 were excluded because they were meta-analyses, reviews or systematic reviews, only clinical trials and controlled trials were included. A total of 18 papers were eligible for review. After evaluating the titles and abstracts, 15 articles were excluded because they were not relevant to the topic of the review [18].

This study is a narrative review designed to provide an overview of the current literature on treatment options for endometriosis-related infertility. To make our work more structured, while still remaining a narrative review, we conducted a PubMed search, reported according to the ‘PRISMA statement’, to search the available literature on the topic. We used the search string “endometriosis-related infertility AND treatment”. A total of 1035 articles were found. We selected only those articles for which free full text was available, and after excluding reviews, meta-analyses and systematic reviews, the total number of eligible articles was 18. Two independent reviewers (A.G. and I.C.) carefully selected the relevant articles after careful evaluation of the title and abstract. Both reviewers determined that only 3 articles met the search criteria. The flow chart and methodology used for the literature review are shown in Figure 1.

This figure shows that there are few studies comparing first-line surgery with IVF in patients with endometriosis-associated infertility. We therefore decided to conduct a narrative review of the data in the literature to provide an overall view of possible treatment strategies, differentiated according to the different types of endometriosis sites. For this reason, we conducted an electronic literature search to evaluate the existing data on the main treatment options, focusing on surgery, for endometriosis-related infertility and to identify common features in the literature to guide therapeutic decisions. The search was conducted using the MEDLINE online medical database (accessed via PubMed). The following keywords were selected and used to filter the literature: “Endometriosis” (MeSH Unique ID: D004715); “Infertility” (MeSH Unique ID: D007246); “Surgical treatment”; “Fertilization in Vitro” (MeSH Unique ID: D005307). We included eligible original articles (randomized and non-randomized clinical trials, prospective observational studies, retrospective cohort studies and case–control studies) and review articles from inception to March 2024. The titles and abstracts of the articles were carefully examined to select those relevant to our research question. We also conducted a thorough review of the bibliographies of the selected articles to identify additional articles to be included. All selected articles were screened by five independent reviewers (I.C., A.G., G.C., G.S., C.D.) who carefully assessed the content for relevance and scientific merit. In total, 72 articles were included for the purposes of our narrative review, i.e., an overview of the available literature on possible treatments for endometriosis-related infertility.

## 3. From Assessment to Management

### 3.1. First Step: Recognizing the Type of Endometriosis

Endometriosis is a very heterogeneous disease, and the treatment should be tailored to the patient’s objectives and based on a specific diagnosis. Transvaginal ultrasound (TVS) examination of the pelvis is now widely accepted as the first-line diagnostic imaging technique. According to the International Deep Endometriosis Analysis (IDEA) group, patients should be systematically evaluated [19]. Pelvic endometriosis can be tricky to be diagnosed because, besides the classical cystic ovarian localization, is it possible it has a blurry ipoechoic lesion involving the anterior and/or the posterior compartment, distortion of the fallopian tubes by the direct presence of the disease or by adhesions and it can also be settled in parametria which can be difficult to study with ultrasound and require considerable experience. As the location of the lesions and the presence of symptoms will guide the choice of treatment, a thorough history must be taken and an accurate TVS performed to map the disease. A key issue is that endometriosis is rarely confined to a single site, but more commonly involves multiple structures, and it is difficult to determine the independent impact on fertility of each localization. Furthermore, adenomyosis may be associated with and contribute to the symptomatology [6,20].

#### 3.1.1. Anterior Compartment

Among the localizations of endometriosis in the anterior compartment, the bladder is the most common, occurring in 84–90% of cases. Since bladder lesions have an extrinsic growth that infiltrates the detrusor muscle from outside rarely reaching the bladder lumen, the treatment of choice is laparoscopic excision of the lesion [21]. Although there is a paucity of data in the literature, some studies have shown that bladder nodules have a negative effect on fertility. Centini et al. conducted a retrospective multicenter study on women with anterior compartment endometriosis associated with otherwise unexplained primary infertility and showed that after bladder surgery, 47.2% of patients conceived during a 2-year follow-up period, of whom 65.4% were spontaneously pregnant [21]. In addition, complete resolution of symptoms was achieved in 84.2% of cases, demonstrating that surgical treatment provides excellent results in terms of both symptoms and infertility [22]. The authors suggest that impaired fertility may be due to changes in the intraperitoneal milieu, which may disrupt ovarian function. In addition, anatomical distortion may lead to fixation of the uterus, contributing to the adverse reproductive outcome. Encouraging data on improved fertility in patients undergoing surgical treatment for bladder injury were also reported by Soriano et al. [23], who showed that 47% of patients treated laparoscopically conceived spontaneously and concluded that surgical treatment should always be offered to symptomatic patients with bladder endometriosis who wish to conceive. They also showed that in a subgroup of 11 patients with previous IVF failure before surgery, six patients had a subsequent pregnancy by IVF and two had a spontaneous pregnancy, suggesting a positive role of surgery also before assisted reproductive techniques (ARTs). It is important to remember that isolated endometriosis of the bladder is a rare condition, more commonly associated with other localizations, particularly deep infiltrating endometriosis (DIE) of the posterior compartment [24].

Timoh et al. [25] reported an overall pregnancy rate of 68% after surgical treatment among women who wished to conceive in the study group, more specifically 70.5% had a spontaneous pregnancy while 29.5% conceived through ART. Among women with previously documented infertility, 52.9% conceived after surgery. The authors showed a high and similar pregnancy rate both in patients with isolated bladder endometriosis and in patients with bladder endometriosis and associated DIE [25], suggesting a positive effect of surgery on fertility in both cases. The same data were reported by Saavalainen et al. [26], who observed a similar pregnancy rate between women treated for bladder endometriosis alone (67%) and women treated for associated DIE (62%).

#### 3.1.2. Posterior Compartment

The posterior compartment is the most frequently affected by endometriotic implants in their various form with an enormous variability across studies [27]. However, prevailing estimates suggest that only 20–40% of patients with endometriosis manifest symptoms related to the disease. Notably, approximately 80% of these lesions are localized within the cul-de-sac area. In 2012, Dai Yi et al. conducted a study regarding the anatomical distribution of pelvic deep infiltrating endometriosis (DIE). Their findings revealed a substantial majority, up to 98.41%, of the deep infiltrative lesions within the posterior pelvic compartment, specifically the uterosacral ligaments (USLs) for 67.08%, followed by the cul-de-sac (12.02%), rectovaginal septum (12.66%), rectum and rectosigmoid junction (2.85%), and ureter (3.80%) [28,29].

In a study conducted by Dückelmann et al. [30], among infertile women treated laparoscopically for DIE, 62.07% achieved pregnancy after surgery, with the majority conceiving spontaneously and only a minority requiring assisted reproductive technology (ART). Similar data were confirmed by Abesadze et al., who found that 43% of patients became pregnant within a median of 6 months, with 50% of pregnancies being spontaneous and resulting in live births [27]. It is essential to emphasize that endometriotic lesions determine the presence of inflammatory mediators, cytokines, growth factors, and immune cells into the peritoneal cavity, creating a complex inflammatory milieu, characterized by increased oxidative stress, angiogenesis, and fibrosis. This altered peritoneal microenvironment not only contributes to the pathogenesis and progression of endometriosis but also presents challenges in its diagnosis and treatment of specific peritoneal endometriotic lesions [31] suggesting a possible beneficial role of surgery [32].

According to Adamson et al.’s meta-analysis, surgical excision of lesions in women with ASRM (American Society for Reproductive Medicine) stage III–IV (stage III corresponding to moderate endometriosis, with 16 to 40 points, and stage IV corresponding to severe endometriosis, with more than 40 points) endometriosis significantly increased the spontaneous pregnancy rate from 4% preoperatively to 43% postoperatively [33,34]. On the other hand, as repeated surgery has a detrimental impact on fertility and should be avoided, when possible, IVF is a viable option in women with deeply infiltrating endometriosis, both as first-line or in women previously operated on. However, studies comparing pregnancy rates after IVF, with or without prior surgery, yield conflicting results and currently do not allow for a definitive conclusion regarding the efficacy of surgical management of deep lesions before IVF. Studies focusing on the spontaneous fertility of infertile patients with deeply infiltrating endometriosis have reported spontaneous pregnancy rates of approximately 10% [35,36] and these data support the idea that surgery does not improve ART [37,38].

As reported above, data are also conflicted regarding IVF, and ENDORE RCT demonstrated, in fact, that pregnancies are achievable after surgery for deep endometriosis, with an overall pregnancy rate exceeding 80% and a spontaneous pregnancy rate over 50%. Among infertile patients who had failed to conceive for more than 1 year preoperatively, 74% achieved pregnancy, with 53% being natural conceptions. This suggests that surgery may eliminate the need for ART in one out of two women while also addressing symptoms [39].

These contrasting opinions are also present in the guidelines. The recent update of the ESHRE 2022 guidelines recommends operative laparoscopy as a therapeutic option for symptomatic women desiring pregnancy, although there is no strong evidence from RCTs that operative laparoscopy enhances fertility outcomes in patients with deep endometriosis [33,40] and states that surgery is necessary if there is bowel or urinary obstruction or if severe pain persisting despite medical treatment [33]; however, it can cause lifelong complications and adhesions that might impair natural or assisted pregnancy success [41]. The guidelines development group (GDG) recommends considering factors such as symptoms, prior surgery history, patient age and preferences, ovarian reserve, other infertility factors, duration of infertility, and the endometriosis fertility index (EFI) when deciding on surgery [33]. The optimal treatment for asymptomatic patients primarily concerned with fertility remains uncertain; first-line IVF, without surgery, seems to be a valid choice, increasingly accepted, in asymptomatic patients. Understanding the impact of surgery on fertility in deep endometriosis is crucial, as evidence suggests surgery could benefit those desiring pregnancy [42].

The approach of first-line surgery and then in vitro fertilization (IVF) is still a possible option: the need for intervention seems to be widely shared in case of persistent infertility and when symptoms significantly affect the quality of life, also causing sexual dysfunction, which is common in cases of posterior compartment involvement [30,43].

In the provision of future pregnancy care in these women, it is important to consider the positive role of surgery in reducing possible adverse obstetric outcomes, but the literature data are also conflicting in this case. Comparative studies show no significant improvement in pregnancy outcomes post-surgery, with some data suggesting increased risks of adverse outcomes such as placenta praevia, caesarean delivery, obstetric hemorrhage, gestational hypertensive disorders, preterm birth, and small for gestational age infants. The role of the endometriosis phenotype and surgical extent in these outcomes is unclear. Moreover, the frequent use of ART in women with endometriosis might further increase obstetric risks [44]. Conversely, Ono et al. found that pregnancy within two years after laparoscopic surgery for endometriosis reduces the risk of placenta previa (2.4% vs. 20%) [45]. The pregnancy outcomes in patients with anterior and posterior compartment endometriosis following surgery for infertility are summarized in Table 1.

#### 3.1.3. Ovarian Endometriosis

Ovarian endometriosis can manifest as endometriomas (OMAs) or superficial implants on the ovarian surface. OMAs are found in up to 44% of women with endometriosis. As explained above, they can be the reason for infertility both directly, through anatomical damage, and through indirect effects such as immunological alterations [46]. Indeed, severe endometriosis connected with the presence of endometriomas appears to have significantly lower pregnancy rates following IVF treatment when compared with severe endometriosis but without endometriomas [47,48].

Surgery has traditionally been the primary treatment option for ovarian endometrioma-related infertility [49]. In infertile women with stage III/IV endometriosis, according to the American Fertility Society/American Society for Reproductive Medicine (AFS/ASRM), operative laparoscopy, instead of expectant management, can be considered to increase spontaneous pregnancy rates [50], but the current evidence shows no benefit of the treatment of OMA associated infertility. The operative laparoscopy prior to ART may increase the chance of natural pregnancy, but surgery has a negative impact on ovarian reserve, evaluated through anti-Müllerian hormone (AMH) levels [33]. Various histological studies confirm that cystectomy is generally associated with the accidental removal of healthy ovarian tissue [2]. Coelho et al. found that ovarian reserve plays a more relevant role than the presence of endometriosis alone as a predictor of fertility outcomes: the chance of conceiving by IVF is similar to that of women without endometriosis when both groups have reduced ovarian reserve (i.e., AFC ≤ 6) [51].

Surgical treatment options include drainage and coagulation/ablation of the cyst, simple drainage of the cyst and excision of the cyst wall. Cyst excision is associated with lower reoperation risk than fenestration and ablation of the cyst wall and improvements in dysmenorrhea, deep dyspareunia, and non-menstrual pain. A meta-analysis of five randomized controlled studies evaluating laparoscopic treatment of endometriosis compared with diagnostic laparoscopy without treatment reported that pain was significantly improved in the treatment group. However, with cyst excision, there is concern about the risk of ovarian damage. A meta-analysis of eight studies of ovarian cystectomy for OMAs found significantly lower anti-Müllerian hormone levels postoperatively [52]. According to two recent meta-analyses, women who underwent cystectomy before IVF/ICSI had a similar live birth rate, clinical pregnancy rate, miscarriage rate, number of oocytes retrieved, and cancellation rate per cycle compared with those with untreated OMA. Therefore, the actual guidelines recommended surgery for OMA prior to ART only to improve endometriosis-associated pain or accessibility of follicles [33,53]. Despite this, operative laparoscopy could be offered as a treatment option for endometriosis-associated infertility in rASRM stage I/II endometriosis, because early diagnosis and treatment of OMAs increase the chances that fibrosis is limited, and vascularization of the ovarian bed is preserved; surgery in an early stage on smaller cysts will cause less ovarian trauma. In women wishing to conceive, conservative surgical management of OMA is always preferred, and several techniques have been studied, ranging from cystectomy by stripping, ablative approaches by laser, plasma energy or bipolar diathermy, sclerotherapy with ethanol and combined approaches. The stripping technique consists of gently pulling apart the cystic wall and it is generally followed by bipolar diathermy haemostasis or suturing of the cyst bed, while ablative techniques require the fenestration, drainage, and destruction of the cyst, more often determining a detrimental effect on ovarian reserve. Laser and plasma energy appear to be more tissue-sparing than bipolar diathermy. Several reports suggest that vaporization or fenestration is favorable compared with cystectomy for OMAs [54]. Patients should be informed of all aspects related to different surgical techniques and both the potential risks of decreased ovarian reserve and possible recurrence.

A systematic review and meta-analysis, which included observational studies and randomized clinical trials, observed a significant decrease in post-operative AMH in bilateral OMAs compared with unilateral OMAs [55,56]. These findings are described also by other groups, like Hirokawa et al. that confirmed the bilaterality of OMAs and the severity of endometriosis are significantly related to the rate of decline in the serum AMH level [57]. Finally, laparoscopic surgery negatively affects the ovarian reserve, and bilateralism and size are compounding adverse factors.

Therefore, surgical planning must be individualized according to patient symptoms and reproductive objectives. The decision to proceed with surgery or not should be considered carefully in patients with a previous ovarian operation, in cases of pre-existing low ovarian reserve or bilateral ovarian OMA.

#### 3.1.4. Other Localizations: Peritoneal Endometriosis and Parametrial Endometriosis

Peritoneal endometriosis (PE) is characterized by the presence of tissue resembling endometrium on the peritoneal surface, even when not macroscopically visible. This tissue typically consists of endometrial stroma. PE lesions are defined as extensions of 5 mm or less beneath the visceral or parietal pelvic peritoneum, non-detectable with imaging process [58]; these lesions exhibit various macroscopic appearances such as clear, black-brown, or orange-red [59]. Analyzing data from the current literature, numerous studies report a correlation between PE and infertility. Reis et al., in their retrospective cross-sectional study, compared the clinical characteristics of patients with histologically confirmed PE to endometriosis-free patients (203 vs. 1292), reporting a significant difference in terms of primary infertility rate (33.2% vs. 18.3%), likely linked to the inflammatory state of the disease affecting both spontaneous fertility and ovulation [60]. Under normal physiological conditions, the peritoneum acts as an immune barrier; however, in cases of PE, this barrier system appears less stable, allowing implantation of ectopic cells during retrograde menstruation [61]. Riccio et al. emphasize the fundamental role played by the inflammatory system in the genesis of PE, reporting decreased reactivity of T cells and NK cytotoxicity, polyclonal activation of B cells, increased antibody production, increased number and activation of peritoneal macrophages, and changes in inflammation mediators underlying the disease presentation [62]. Vallvé-Juanico et al., in their study evaluating the immune environment in endometriosis patients, report a close association between the disease and the macrophage cell population [63]. At the endometrial and peritoneal levels in endometriosis patients, the concentration of macrophages in its subpopulations does not follow normal menstrual cycle fluctuations, and this stable concentration predisposes increased cell survival, predisposing ectopic lesion formation through migration and creating an inhospitable environment for pregnancy [64]. PE activates numerous pathways leading to continuous leukocyte recruitment, resulting in a state of chronic inflammation. Inflammatory mediators such as IL-8, TNF-alpha, and intercellular adhesion molecules (ICAMs) contribute to fallopian tube distortion through the mechanical effects of pelvic adhesions. In addition to a mechanically mediated effect from adhesions, the altered peritoneal environment also affects tubal ciliary beat frequency, further reducing fertility [64]. Tanbo et al. assert that PE, with its inflammatory state, negatively influences ovulation, compromises oocyte quality, and impedes fertility by affecting endometrial decidualization and subsequent embryo implantation [65].

In summary, peritoneal endometriosis represents a complex interplay between immune dysregulation, chronic inflammation, and reproductive pathology, characterized by the presence of tissue resembling endometrium on the peritoneal surface and its associated effects on fertility. The role of surgical treatment for peritoneal endometriosis (PE) remains a topic of debate. Gale advocates for in vitro fertilization (IVF) as the first-line treatment for patients with infertility related to endometriosis, reserving surgery only for selected cases with debilitating symptoms affecting quality of life, neoplastic risk, organ obstruction, or dysfunction [66]. ASRM, ESHRE, and SGOC consider surgery as an option to be offered to women with infertility and suspected mild or moderate endometriosis following careful evaluation of patient age, symptoms, and subsequent fertility treatment options (IVF, clomiphene, gonadotropins [67]. However, all societies emphasize that surgery should not be performed in asymptomatic women with infertility to search for signs of endometriosis [33]. Regarding the type of surgical approach to PE, Riley et al. conducted a randomized surgical trial comparing surgical excision versus ablation of PE in symptomatic patients, and the data show no long-term differences in terms of dysmenorrhea, dyschezia, and dyspareunia [68]. Rolla suggests that peritoneal lesions should be radically removed up to extensive peritonectomies in symptomatic patients because they often conceal underlying deep endometriosis and allow for histological evaluation not feasible with destructive techniques (electrocoagulation or laser [69]. Currently, laparoscopy is widely used in infertility associated with endometriosis. However, it should be noted that the outcome of spontaneous pregnancy after surgery depends on the presence of tubal adhesions. In cases of mild endometriosis such as PE, laparoscopy is an effective approach to remove any tubal adhesions, but alternative IVF-ET treatment may be equally valid because it is not influenced by the presence of tubal adhesions [70]. Personalization of treatment is essential in cases of PE associated with infertility, considering symptoms, disease impact on quality of life, and patient desire, and resorting to surgery only when strictly necessary.

Another possible location of endometriosis that may contribute to infertility but is less studied is the parametrium. The parametrium consists of a connective tissue mesentery primarily composed of areolar tissue that envelops the visceral branches of the hypogastric vessels during their course toward the uterus, with the deep uterine vein serving as the main anatomical landmark for pelvic autonomic nerves. Dissections have shown that these tissue masses can be arbitrarily distinguished into different structures based on the number and types of vascular pedicles found in each sample [58]. Conventionally, tissues crossing above the ureter are identified as parametrium, while those crossing below are considered paracervix [59].

Parametrial endometriosis (PaE) is encountered in advanced disease scenarios, where stenosis and dilatation of the ureter and voiding dysfunctions are often observed due to the involvement of the inferior hypogastric plexus [60]. The localization of parametrial disease can be accurately studied using transvaginal ultrasound (TVS [59]) or magnetic resonance imaging (MRI [57]). Regarding treatment, the efficacy of medical therapy for PaE is not known in the current literature; however, numerous studies have investigated surgical treatment and its associated challenges [63]. With a radical approach to endometriosis, parametrectomy significantly impacts operative time and intraoperative and postoperative morbidity due to potential damage to the sympathetic and parasympathetic fibers of the inferior hypogastric plexus, leading to neurogenic pelvic dysfunctions. Benoit et al. analyzed complication rates in a retrospective study of 753 patients undergoing surgery for deep infiltrating endometriosis (DIE), dividing subjects into those with and without PaE (37.8% vs. 62.2%); the data highlighted higher rates of postoperative adverse events, particularly voiding dysfunctions, in patients undergoing parametrectomy [64]. Mabrouk et al. evaluated 1360 patients undergoing surgery for DIE, finding a prevalence of PaE of 17% and confirming higher rates of urinary dysfunction requiring self-catheterization postoperatively in patients undergoing parametrectomy; the data indicated that the presence of PaE did not affect infertility outcomes [71]. Currently, there are no studies primarily evaluating the impact of parametrial endometriosis on fertility; however, given that PaE is a severe form of DIE, it could negatively impact oocyte production, ovulation, fertilization, and pregnancy implantation due to its inflammatory component [65]. In the systematic review and meta-analysis by Casals et al. on the impact of surgery for DIE prior to IVF, surgical treatment was found to improve IVF outcomes in terms of positive pregnancy rates (PRp), clinical pregnancy rates (PRc), and live birth rates (LBRp) compared to patients undergoing direct IVF. However, this finding should be validated by randomized controlled trials (RCTs) with sufficient power and follow-up to confirm and appropriately quantify the benefits and risks of surgical intervention prior to IVF [66].

Currently, in cases of PaE associated with infertility, it is not possible to establish superiority in terms of efficacy between surgical treatment and IVF.

### 3.2. Second Step: Choose the Tailored Approach and the Correct Timing (When and Why?)

**Figure 2:**
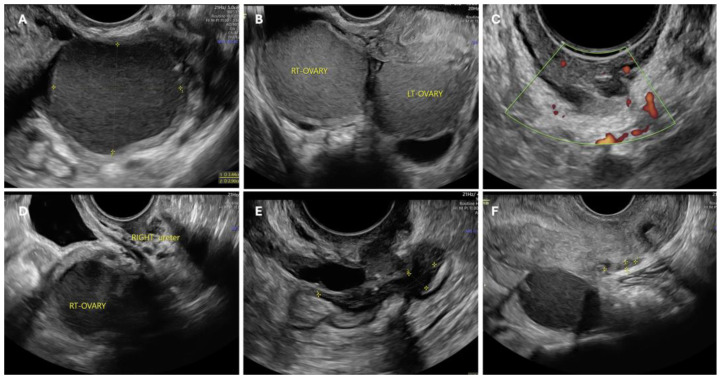
Different ultrasonographic cases of pelvic endometriosis. Ovarian involvement may be unilateral (**A**) or bilateral (**B**), giving the characteristic ‘kissing ovaries’ sign when the two affected ovaries are attached posteriorly to the uterus. Parametrial endometriosis, nodule involving the right ureter (**C**). It is important to note that pelvic structures are closely located and therefore it is common for adjacent structures to be infiltrated or retracted by endometriosis, as in the case of the ureter (**D**,**F**). Figure (**E**) shows a case of endometriosis of the anterior wall of the rectum. Often the lesions are multiple, resulting in complex presentations with possible involvement of the ovary, uterosacral ligaments, and rectum, resulting hypomobility of the structures, Douglas’ obliteration and even frozen pelvis.

**Figure 3:**
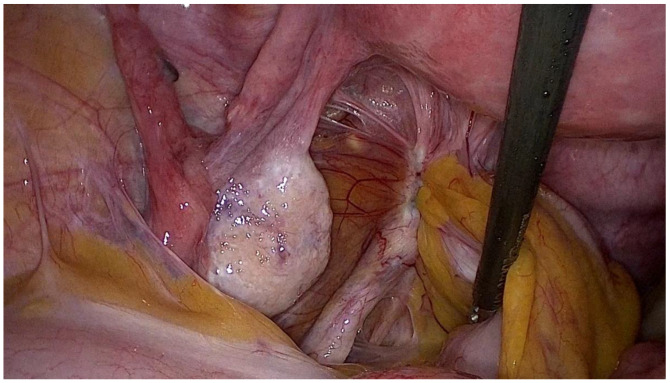
Laparoscopic view of endometriosis of the posterior compartment involving the left uterosacral ligament and the rectum.

Endometriosis is often associated with infertility, furthermore, patients can experience unbearable pain requiring therapy to relieve symptoms. Unfortunately, the hormonal nature of the disease leads to undesirable contraceptive effects, making most therapies unsuitable for women wishing to conceive. Thus, the possible treatments for endometriosis-related infertility are surgery, IVF, or a combination of the two, but in the absence of specific guidelines, decisions in these cases often depend on the physician’s preference [72]. The first step in choosing a personalized treatment is to assess the presence of symptoms and the location and extent of lesions along with the patient’s requirements. Some ultrasound images of infertile patients with different locations of endometriosis lesions are shown in Figure 2. From a pro-surgery view, it seems that symptomatic women with mild disease and unexplained infertility may benefit from surgical treatment, with better reproductive outcomes compared to untreated women [73]. In addition, surgery should also be considered as a first-line option in the case of voluminous OMAs with doubtful ultrasonographic appearance [74]. It may also increase the chances of subsequent IVF by improving follicular access [50] and preventing cyst rupture during pregnancy [72] but in the case of small OMAs, literature reviews have shown that surgical treatment prior to IVF does not increase the chances of pregnancy [75]. The location of the lesions must be considered before planning surgery (Figure 3). In ovarian endometriosis, the presence of the cyst itself determines the reduction in ovarian function, but as explained above, surgical treatment can contribute to damage to the follicular reserve making the choice of treatment a challenge in which risks and benefits must be balanced. As reported above, ovarian reserve can be assessed using a variety of measures, the most commonly employed being anti-Müllerian hormone (AMH) and ultrasound antral follicle count (AFC) [76]. AHM is produced by the granulosas cell and regulates the number of primordial follicles maturing, it is an objective measure but not yet standardized and it is still unknown its value in the case of monolateral ovarian surgery. AFC is the measurement of the number of follicles with a diameter of 2–10 mm, easy to perform by transvaginal ultrasound that clearly shows the follicular activity both in the operated and non-operated ovary, but on the other hand, it shows intra- and inter-cycle variation and it is operator dependent [76]. When using AMH as a marker of ovarian function in endometriosis, we have to consider that OMAs per se reduce its value and AFC may be reduced in the affected ovary due to poor visualization of the follicles because of the presence of the cyst or an intrinsic reduction in the follicles as a result of the pathology [76]. For all these reasons, the role of preoperative and postoperative AMH and AFC is still unclear. Traditionally, cystectomy has been the preferred technique because of a reduced risk of recurrence, but it is associated with a higher risk of reduced ovarian volume and thus ovarian function [76]. Being a skilled surgeon is not always enough to treat endometriosis-related infertility, better results are only provided in the case of extensive experience of a “dedicated endometriosis” surgeon who is able to assess how radical to treat the disease, restore the pelvic anatomy and avoid ovarian or tubal injury [77].

**Figure 4:**
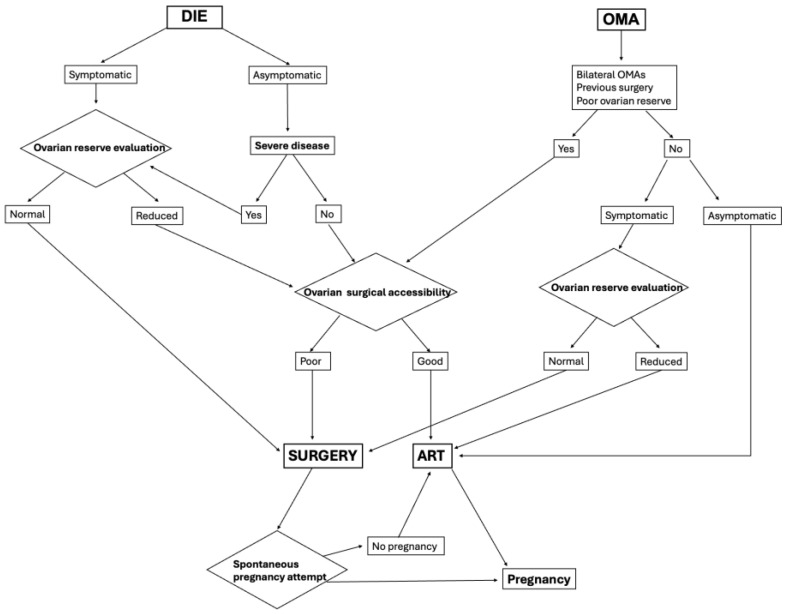
Flowchart for the management of infertile patients in relation to the location of lesions and the presence of symptoms.

Repeated ovarian surgery has a detrimental effect on ovarian function and reduces the chances of pregnancy; therefore, the decision to undergo surgical treatment should be delayed until pregnancy is desired, and surgery should be performed in a referral center. Centini et al. [76] demonstrated an increased pregnancy and live birth rate in young women surgically treated for endometriosis with a beneficial effect more evident when it was the first intervention. The authors highlighted how the radicality of treatment is still under debate as extensive surgery can be linked to post-operative complications which may be related also to poorer reproductive and obstetric outcomes. Counseling before surgical intervention is fundamental as women must know that the best time to conceive is within the first year after surgery and for this reason, the timing of treatment must be tailored according to women’s reproductive desire [76]. Since laparoscopy is no longer considered the gold standard for the diagnosis of endometriosis, surgical treatment should be reserved for cases of confirmed infertility, in symptomatic patients, or in patients in whom large lesions could undergo decidualization with consequent complications during pregnancy. From a pro-IVF point of view, surgical treatment should be ruled out in the case of previous surgery and bilateral OMAs because of a high risk of severe impairment of ovarian reserve [72]. IVF should also be considered first in cases of male infertility or advanced age with reduced ovarian reserve, to minimize the time to pregnancy and the depletion of ovarian function [77]. Each treatment option has pros and cons. Surgery carries a high risk of recurrence, reduced ovarian reserve and surgical risks, while IVF carries a risk of ovarian abscess, ovarian hyperstimulation syndrome and multiple pregnancies [72]. Choosing the right time for surgery or IVF is essential to improve the chances of pregnancy. When faced with a patient with endometriosis-related infertility, we must therefore consider the presence of symptoms, the previous surgical history and the location and extent of the disease in order to choose the most appropriate personalized treatment and, above all, we must be able to explain to the patient why we are proposing one treatment rather than another and share the decision [53]. Figure 4 shows a flow chart for the management of infertile patients in relation to the location of lesions and the presence of symptoms.

## 4. Conclusions

Medical centers treating endometriosis-related infertility should offer both surgical and IVF strategies as a similar live birth rate of around 25% is seen with both approaches [72].

When choosing infertility management all causes must be evaluated, surgery should only be employed in symptomatic patients or when all other causes of infertility have been ruled out. IVF is generally preferred in cases of bilateral OMAs or previous pelvic surgery, and it is also a quicker way to conceive making it more feasible for women of advanced age. The benefit of OMA excision for pain management is clear: ovarian cystectomy is the most effective surgical technique for the management of OMA, compared with other excisional/ablative techniques. Surgical excision only to improve fertility outcomes is not supported by the currently available research. For this reason, surgical management should be individualized for women with OMA, remembering that all the techniques cause a reduction in ovarian reserve, expressed by a reduction in AMH. In this individualized management, strong consideration should be given to the preoperative ovarian reserve status prior to performing ovarian cystectomy [47]. A shared and informed decision is essential to achieve the best outcome, and treatment must be tailored considering several factors such as age, ovarian reserve, duration of infertility, concomitant male infertility, extent of disease, previous pelvic surgery, and symptoms.

## Figures and Tables

**Table 1:** Summary of pregnancy outcomes after surgery for infertility in patients with endometriosis of the anterior and posterior compartments.

Location of Lesions	Authors	Pregnancies Outcomes After Surgery
Bladder	Centini et al., 2018 [21]	47.2% of conceptions after surgery, 65.4% spontaneous
	Soriano et al., 2016 [23]	47% of spontaneous conceptions
	Saavalainen et al., 2016 [26]	67% of pregnancies after surgery
Bladder + DIE	Timoh et al., 2018 [25]	Overall pregnancy rate of 68, 70.5% spontaneous conception
	Saavalainen et al., 2016 [26]	62% of pregnancies after surgery
Posterior compartment	Dückelmann et al., 2021 [30]	62.07% of pregnancies after surgery. The majority conceived spontaneously
	Abesadze et al., 2020 [27]	43% of pregnancies after surgery within a median of 6 months, 50% of pregnancies being spontaneous

## Data Availability

Not applicable.

## References

[1] Martire F.G., Giorgi M., D’Abate C., Colombi I., Ginetti A., Cannoni A., Fedele F., Exacoustos C., Centini G., Zupi E. (2024). Deep Infiltrating Endometriosis in Adolescence: Early Diagnosis and Possible Prevention of Disease Progression. J. Clin. Med..

[2] Bonavina G., Taylor H.S. (2022). Endometriosis-associated infertility: From pathophysiology to tailored treatment. Front. Endocrinol..

[3] Centini G., Schettini G., Pieri E., Giorgi M., Lazzeri L., Martire F.G., Mancini V., Raimondo D., Seracchioli R., Habib N. (2024). Endometriosis-Related Ovarian Cancer: Where Are We Now? A Narrative Review towards a Pragmatic Approach. J. Clin. Med..

[4] Taylor H.S., Kotlyar A.M., Flores V.A. (2021). Endometriosis is a chronic systemic disease: Clinical challenges and novel innovations. Lancet Lond. Engl..

[5] Practice Committee of the American Society for Reproductive Medicine (2006). Definition of “infertility”. Fertil. Steril..

[6] Somigliana E., Garcia-Velasco J.A. (2015). Treatment of infertility associated with deep endometriosis: Definition of therapeutic balances. Fertil. Steril..

[7] Matsuzaki S., Schubert B. (2010). Oxidative stress status in normal ovarian cortex surrounding ovarian endometriosis. Fertil. Steril..

[8] Sanchez A.M., Viganò P., Somigliana E., Panina-Bordignon P., Vercellini P., Candiani M. (2014). The distinguishing cellular and molecular features of the endometriotic ovarian cyst: From pathophysiology to the potential endometrioma-mediated damage to the ovary. Hum. Reprod. Update.

[9] Nelson S.M. (2013). Biomarkers of ovarian response: Current and future applications. Fertil. Steril..

[10] Agarwal S.K., Chapron C., Giudice L.C., Laufer M.R., Leyland N., Missmer S.A., Singh S.S., Taylor H.S. (2019). Clinical diagnosis of endometriosis: A call to action. Am. J. Obstet. Gynecol..

[11] Eisenberg V.H., Decter D.H., Chodick G., Shalev V., Weil C. (2022). Burden of Endometriosis: Infertility, Comorbidities, and Healthcare Resource Utilization. J. Clin. Med..

[12] Vercellini P., Meana M., Hummelshoj L., Somigliana E., Viganò P., Fedele L. (2011). Priorities for endometriosis research: A proposed focus on deep dyspareunia. Reprod. Sci. Thousand Oaks Calif..

[13] Denny E., Mann C.H. (2007). Endometriosis-associated dyspareunia: The impact on women’s lives. J. Fam. Plann Reprod. Health Care.

[14] Martire F.G., Piccione E., Exacoustos C., Zupi E. (2023). Endometriosis and Adolescence: The Impact of Dysmenorrhea. J. Clin. Med..

[15] Yong P.J., Sadownik L., Brotto L.A. (2015). Concurrent deep-superficial dyspareunia: Prevalence, associations, and outcomes in a multidisciplinary vulvodynia program. J. Sex. Med..

[16] Mabrouk M., Del Forno S., Spezzano A., Raimondo D., Arena A., Zanello M., Leonardi D., Paradisi R., Seracchioli R. (2020). Painful Love: Superficial Dyspareunia and Three Dimensional Transperineal Ultrasound Evaluation of Pelvic Floor Muscle in Women with Endometriosis. J. Sex. Marital. Ther..

[17] Johnson N.P. (2014). Review of lipiodol treatment for infertility—An innovative treatment for endometriosis-related infertility?. Aust. N. Z. J. Obstet. Gynaecol..

[18] Page M.J., McKenzie J.E., Bossuyt P.M., Boutron I., Hoffmann T.C., Mulrow C.D., Shamseer L., Tetzlaff J.M., Akl E.A., Brennan S.E. (2021). The PRISMA 2020 statement: An updated guideline for reporting systematic reviews. BMJ.

[19] Guerriero S., Condous G., van den Bosch T., Valentin L., Leone F.P.G., Van Schoubroeck D., Exacoustos C., Installé A.J.F., Martins W.P., Abrao M.S. (2016). Systematic approach to sonographic evaluation of the pelvis in women with suspected endometriosis, including terms, definitions and measurements: A consensus opinion from the International Deep Endometriosis Analysis (IDEA) group. Ultrasound Obstet. Gynecol. Off. J. Int. Soc. Ultrasound Obstet. Gynecol..

[20] Cozzolino M., Cosentino M., Loiudice L., Martire F.G., Galliano D., Pellicer A., Exacoustos C. (2024). Impact of adenomyosis on in vitro fertilization outcomes in women undergoing donor oocyte transfers: A prospective observational study. Fertil. Steril..

[21] Centini G., Afors K., Alves J., Argay I.M., Koninckx P.R., Lazzeri L., Monti G., Zupi E., Wattiez A. (2018). Effect of Anterior Compartment Endometriosis Excision on Infertility. JSLS.

[22] Martire F.G., Russo C., Selntigia A., Siciliano T., Lazzeri L., Piccione E., Zupi E., Exacoustos C. (2022). Transvaginal ultrasound evaluation of the pelvis and symptoms after laparoscopic partial cystectomy for bladder endometriosis. J. Turk. Ger. Gynecol. Asso..

[23] Soriano D., Bouaziz J., Elizur S., Zolti M., Orvieto R., Seidman D., Goldenberg M., Eisenberg H.V. (2016). Reproductive Outcome Is Favorable After Laparoscopic Resection of Bladder Endometriosis. J. Minim. Invasive Gynecol..

[24] Wattiez A., Puga M., Albornoz J., Faller E. (2013). Surgical strategy in endometriosis. Best. Pract. Res. Clin. Obstet. Gynaecol..

[25] Nyangoh Timoh K., Ballester M., Bendifallah S., Fauconnier A., Darai E. (2018). Fertility outcomes after laparoscopic partial bladder resection for deep endometriosis: Retrospective analysis from two expert centres and review of the literature. Eur. J. Obstet. Gynecol. Reprod. Biol..

[26] Saavalainen L., Heikinheimo O., Tiitinen A., Härkki P. (2016). Deep infiltrating endometriosis affecting the urinary tract-surgical treatment and fertility outcomes in 2004–2013. Gynecol. Surg..

[27] Abesadze E., Sehouli J., Mechsner S., Chiantera V. (2020). Possible Role of the Posterior Compartment Peritonectomy, as a Part of the Complex Surgery, Regarding Recurrence Rate, Improvement of Symptoms and Fertility Rate in Patients with Endometriosis, Long-Term Follow-Up. J. Minim. Invasive Gynecol..

[28] Dai Y., Leng J.H., Lang J.H., Li X.Y., Zhang J.J. (2012). Anatomical distribution of pelvic deep infiltrating endometriosis and its relationship with pain symptoms. Chin. Med. J..

[29] Chapron C., Fritel X., Dubuisson J.B. (1999). Fertility after laparoscopic management of deep endometriosis infiltrating the uterosacral ligaments. Hum. Reprod. Oxf. Engl..

[30] Dückelmann A.M., Taube E., Abesadze E., Chiantera V., Sehouli J., Mechsner S. (2021). When and how should peritoneal endometriosis be operated on in order to improve fertility rates and symptoms? The experience and outcomes of nearly 100 cases. Arch. Gynecol. Obstet..

[31] Kanno K., Andou M., Aiko K., Yoshino Y., Sawada M., Sakate S., Yanai S. (2021). Fertility- and Nerve-sparing Laparoscopic Eradication of Deep Endometriosis with Total Posterior Compartment Peritonectomy: The Kurashiki Method. J. Minim. Invasive Gynecol..

[32] Martire F.G., Zupi E., Lazzeri L., Morosetti G., Conway F., Centini G., Solima E., Pietropolli A., Piccione E., Exacoustos C. (2021). Transvaginal Ultrasound Findings After Laparoscopic Rectosigmoid Segmental Resection for Deep Infiltrating Endometriosis. J. Ultrasound Med..

[33] Becker C.M., Bokor A., Heikinheimo O., Horne A., Jansen F., Kiesel L., King K., Kvaskoff M., Nap A., Petersen K. (2022). ESHRE guideline: Endometriosis. Hum. Reprod. Open.

[34] Adamson G.D., Pasta D.J. (2010). Endometriosis fertility index: The new, validated endometriosis staging system. Fertil. Steril..

[35] Practice Committee of the American Society for Reproductive Medicine (2012). Endometriosis and infertility: A committee opinion. Fertil. Steril..

[36] Borghese B., Santulli P., Marcellin L., Chapron C. (2018). Definition, description, clinicopathological features, pathogenesis and natural history of endometriosis: CNGOF-HAS Endometriosis Guidelines. Gynecol. Obstet. Fertil. Senol..

[37] Laursen J.B., Schroll J.B. (2019). Letter to the editor. Arch. Gynecol. Obstet..

[38] Khan S., Lee C.L. (2021). Treating Deep Endometriosis in Infertile Patients before Assisted Reproductive Technology. Gynecol. Minim. Invasive Ther..

[39] Roman H., Huet E., Bridoux V., Khalil H., Hennetier C., Bubenheim M., Braund S., Tuech J.-J. (2022). Long-term Outcomes Following Surgical Management of Rectal Endometriosis: Seven-year Follow-up of Patients Enrolled in a Randomized Trial. J. Minim. Invasive Gynecol..

[40] Daniilidis A., Angioni S., Di Michele S., Dinas K., Gkrozou F., D’Alterio M.N. (2022). Deep Endometriosis and Infertility: What Is the Impact of Surgery?. J. Clin. Med..

[41] Vercellini P., Somigliana E., Viganò P., Abbiati A., Barbara G., Crosignani P.G. (2009). Surgery for endometriosis-associated infertility: A pragmatic approach. Hum. Reprod. Oxf. Engl..

[42] Roman H. (2015). Colorectal endometriosis and pregnancy wish: Why doing primary surgery. Front. Biosci. Sch. Ed..

[43] Donnez O. (2021). Conservative Management of Rectovaginal Deep Endometriosis: Shaving Should Be Considered as the Primary Surgical Approach in a High Majority of Cases. J. Clin. Med..

[44] Mooney S.S., Ross V., Stern C., Rogers P.A.W., Healey M. (2021). Obstetric Outcome After Surgical Treatment of Endometriosis: A Review of the Literature. Front. Reprod. Health..

[45] Ono Y., Furumura K., Yoshino O., Ota H., Sasaki Y., Hidaka T., Fukushi Y., Hirata S., Yamada H., Wada S. (2022). Influence of laparoscopic surgery for endometriosis and its recurrence on perinatal outcomes. Reprod. Med. Biol..

[46] Hamdan M., Dunselman G., Li T.C., Cheong Y. (2015). The impact of endometrioma on IVF/ICSI outcomes: A systematic review and meta-analysis. Hum. Reprod. Update.

[47] Cranney R., Condous G., Reid S. (2017). An update on the diagnosis, surgical management, and fertility outcomes for women with endometrioma. Acta Obstet. Gynecol. Scand..

[48] Coccia M.E., Nardone L., Rizzello F. (2022). Endometriosis and Infertility: A Long-Life Approach to Preserve Reproductive Integrity. Int. J. Environ. Res. Public. Health.

[49] Cecchino G.N., García-Velasco J.A. (2018). Endometrioma, fertility, and assisted reproductive treatments: Connecting the dots. Curr. Opin. Obstet. Gynecol..

[50] Dunselman G.A.J., Vermeulen N., Becker C., Calhaz-Jorge C., D’Hooghe T., De Bie B., Heikinheimo O., Horne A.W., Kiesel L., Nap A. (2014). ESHRE guideline: Management of women with endometriosis. Hum. Reprod..

[51] Neto M.A.C., Martins W.d.P., Luz C., Jianini B.T.G.M., Ferriani R.A., Navarro P.A. (2016). Endometriosis, Ovarian Reserve and Live Birth Rate Following In Vitro Fertilization/Intracytoplasmic Sperm Injection. Rev. Bras. Ginecol. Obstet. RBGO Gynecol. Obstet..

[52] Practice Committee of the American Society for Reproductive Medicine (2014). Treatment of pelvic pain associated with endometriosis: A committee opinion. Fertil. Steril..

[53] Centini G., Afors K., Murtada R., Argay I.M., Lazzeri L., Akladios C.Y., Zupi E., Petraglia F., Wattiez A. (2016). Impact of Laparoscopic Surgical Management of Deep Endometriosis on Pregnancy Rate. J. Minim. Invasive Gynecol..

[54] Var T., Batioglu S., Tonguc E., Kahyaoglu I. (2011). The effect of laparoscopic ovarian cystectomy versus coagulation in bilateral endometriomas on ovarian reserve as determined by antral follicle count and ovarian volume: A prospective randomized study. Fertil. Steril..

[55] Moreno-Sepulveda J., Romeral C., Niño G., Pérez-Benavente A. (2022). The Effect of Laparoscopic Endometrioma Surgery on Anti-Müllerian Hormone: A Systematic Review of the Literature and Meta-Analysis. JBRA Assist. Reprod..

[56] Mehdizadeh Kashi A., Chaichian S., Ariana S., Fazaeli M., Moradi Y., Rashidi M., Najmi Z. (2017). The impact of laparoscopic cystectomy on ovarian reserve in patients with unilateral and bilateral endometrioma. Int. J. Gynecol. Obstet..

[57] Hirokawa W., Iwase A., Goto M., Takikawa S., Nagatomo Y., Nakahara T., Bayasula B., Nakamura T., Manabe S., Kikkawa F. (2011). The post-operative decline in serum anti-Mullerian hormone correlates with the bilaterality and severity of endometriosis. Hum. Reprod. Oxf. Engl..

[58] Kiesel L., Sourouni M. (2019). Diagnosis of endometriosis in the 21st century. Climacteric.

[59] Tomassetti C., Johnson N.P., Petrozza J., Abrao M.S., Einarsson J.I., Horne A.W., Lee T.T.M., Missmer S., Vermeulen N., Zondervan K.T. (2021). An international terminology for endometriosis, 2021. Hum. Reprod. Open.

[60] Reis F.M., Santulli P., Marcellin L., Borghese B., Lafay-Pillet M.C., Chapron C. (2020). Superficial Peritoneal Endometriosis: Clinical Characteristics of 203 Confirmed Cases and 1292 Endometriosis-Free Controls. Reprod. Sci. Thousand Oaks Calif..

[61] Holl M., Becker L., Keller A.L., Feuerer N., Marzi J., Carvajal Berrio D.A., Jakubowski P., Neis F., Pauluschke-Fröhlich J., Brucker S.Y. (2021). Laparoscopic Peritoneal Wash Cytology-Derived Primary Human Mesothelial Cells for In Vitro Cell Culture and Simulation of Human Peritoneum. Biomedicines.

[62] Da Gama Coelho Riccio L., Santulli P., Marcellin L., Abrão M.S., Batteux F., Chapron C. (2018). Immunology of endometriosis. Best. Pract. Res. Clin. Obstet. Gynaecol..

[63] Vallvé-Juanico J., Houshdaran S., Giudice L.C. (2019). The endometrial immune environment of women with endometriosis. Hum. Reprod. Update.

[64] Khan K.N., Masuzaki H., Fujishita A., Kitajima M., Sekine I., Ishimaru T. (2004). Differential macrophage infiltration in early and advanced endometriosis and adjacent peritoneum. Fertil. Steril..

[65] Tanbo T., Fedorcsak P. (2017). Endometriosis-associated infertility: Aspects of pathophysiological mechanisms and treatment options. Acta Obstet. Gynecol. Scand..

[66] Gale J., Singh S.S. (2022). A Practical Approach to Fertility Considerations in Endometriosis Surgery. Obstet. Gynecol. Clin. North. Am..

[67] Kho R.M., Andres M.P., Borrelli G.M., Neto J.S., Zanluchi A., Abrão M.S. (2018). Surgical treatment of different types of endometriosis: Comparison of major society guidelines and preferred clinical algorithms. Best. Pract. Res. Clin. Obstet. Gynaecol..

[68] Riley K.A., Benton A.S., Deimling T.A., Kunselman A.R., Harkins G.J. (2019). Surgical Excision Versus Ablation for Superficial Endometriosis-Associated Pain: A Randomized Controlled Trial. J. Minim. Invasive Gynecol..

[69] Rolla E. (2019). Endometriosis: Advances and controversies in classification, pathogenesis, diagnosis, and treatment. F1000Research.

[70] Osuga Y., Koga K., Tsutsumi O., Yano T., Maruyama M., Kugu K., Momoeda M., Taketani Y. (2002). Role of laparoscopy in the treatment of endometriosis-associated infertility. Gynecol. Obstet. Investig..

[71] Wu M.Y., Ho H.N. (2003). The role of cytokines in endometriosis. Am. J. Reprod. Immunol..

[72] Lessey B.A., Gordts S., Donnez O., Somigliana E., Chapron C., Garcia-Velasco J.A., Donnez J. (2018). Ovarian endometriosis and infertility: In vitro fertilization (IVF) or surgery as the first approach?. Fertil. Steril..

[73] Marcoux S., Maheux R., Bérubé S. (1997). Laparoscopic surgery in infertile women with minimal or mild endometriosis. Canadian Collaborative Group on Endometriosis. N. Engl. J. Med..

[74] Somigliana E., Benaglia L., Paffoni A., Busnelli A., Vigano P., Vercellini P. (2015). Risks of conservative management in women with ovarian endometriomas undergoing IVF. Hum. Reprod. Update.

[75] ETIC Endometriosis Treatment Italian Club (2019). When more is not better: 10 «don’ts» in endometriosis management. An ETIC * position statement. Hum. Reprod. Open.

[76] Daniilidis A., Grigoriadis G., Kalaitzopoulos D.R., Angioni S., Kalkan Ü., Crestani A., Merlot B., Roman H. (2023). Surgical Management of Ovarian Endometrioma: Impact on Ovarian Reserve Parameters and Reproductive Outcomes. J. Clin. Med..

[77] Gizzo S., Conte L., Di Gangi S., Leggieri C., Quaranta M., Noventa M., Litta P., Saccardi C. (2015). Could surgeon’s expertise resolve the debate about surgery effectiveness in treatment of endometriosis-related infertility?. Arch. Gynecol. Obstet..

